# Supplementary material for: Chromothripsis during telomere crisis is independent of NHEJ, and consistent with a replicative origin
Source: Genome Res. 2019 May;29(5):737–49. doi: 10.1101/gr.240705.118 (PMC6499312; doi:10.1101/gr.240705.118)
Supplement: Supplemental Material [file supp_gr.240705.118_Supplemental_file_1.zip › contigs/annotated_contigs/DB57/contig.3.DB57_length_1264_mean_cov_19.8291139241.docx]

**DB57_length_1264_mean_cov_19.8291139241**

```
AGGCAGGAGAATCACTTGAACCCGGGAGGCAGAGGTTGCAGTGAGCCGATTGTGCCACTGCACTCCAGCATGGGGGACAGAGTGAGACT
 >chr11:47161126-47161749 + E=0e+00
GAACGTCAAAAAAAAAAGGGAGCATGAAAGTGATGAACCCCAGTTGAGGGAAGGGTACTGTTACTGCTCACTTCACTGAAGTGGGAACT

GAGGCTCAGACGGGTGACAATGTTGTGCGAATATACTGATAGCTTGTAAGTAGCAGTGGCAGAACTGGAACCGCAGTCAGCCTGAATCC

AGAGATGGGGGATCGGGAGCACAAGGAGTCTCTTCATGCTCCCACCTCCCTGTGTCATGGCATCATTTCTGCTTTGTGGTTTGACCCAC

TGAAAACAAAGCTGCATTGGCTGCACTGCACCAAGAATCACCTCGCTCCTTTTGAAATGTGCCAACCAGCTCCTCTATTGACTTGGAAG

ATGGGAACTCCATTGCTCCCAGTGACAGACACCAGCCTCACGGTGGGGCACGGTGTTTTGCAGCTAACAGCTCATGACCTACTTTTGCT

GGTCATGTGCAAAACCTGTTTAGTGATCACACATGCAAAATTTTGTAGGTTTTTTTCTCCCTCATTATTCAGTGGATAATT|TTC|AAG
                                                                                  >chr1
TGATTCTCCTGCCTTAGCCTCCTGAGTAGCTGGGATTATAGATGTGCACCACCATGCCCAGCTAAGTTTTGTATTTTTAGTAGAGACAG
1:47162971-47163405 - E=3e-239 p=0e+00
GTTTCACCATGTTGGCCAGGCTGGGTTTGAACTCCTGACCTCAAATGATCCTCCTGCTTCAGCCTTCCAAAGTGCTGGGATTACAGGCG

TGAGCCACTATGCCTGGCCAAGACCACATATACAATTCTACTTATTTGATTTTTTTTTAAAGACAGAGTCTCACTCAGTTGCCCAGGCT

GGAGTGCAGTGGCACAATCTCAGCTTACTACAACCTCTGCCCCCCAGGCTCAAGCAATTCTCCCACCTCAGCCTCCCAAGTAATTGGGA

CCACAGACGCACACCACCACGCCTGGCTAAG|TTTTTGTATTTTTGTTAGAGATGGGGTTTCACCATG|ATAGCCAGGATGGTCTCCAT
                                >chr11:47162256-47162500 - E=1e-128
CTCCTGACCTCGTGATCCGCCTGCCTTGGCCTCCCAAAGTGCTGGGATTACAGGCGTGAGCCACCGTGCCCGGCCTTAAGTTCGGTCTT

TAAGGATGCTGAGTTTATCTCCCCAGATTTTGGGGCTGAATATTCCTTAAAACAATCTGGGCCCAGGCTGGCTGTGGTGGCTCATGCCT

GTAATCCCAG|CACTTTGGGAGG
```
